# Horseshoe crab bio-ecological data from Balok, East Coast Peninsular Malaysia

**DOI:** 10.1016/j.dib.2018.12.027

**Published:** 2018-12-13

**Authors:** Nurul Ashikin Mat Zauki, Behara Satyanarayana, Nur Fairuz-Fozi, Bryan Raveen Nelson, Melissa Beata Martin, Bavajohn Akbar-John, Ahmed Jalal Khan Chowdhury

**Affiliations:** a Institute of Oceanography and Environment (INOS), Universiti Malaysia Terengganu, 21030 Kuala Nerus, Terengganu, Malaysia.; b Institute of Tropical Biodiversity and Sustainable Development (Bio-D Tropika), Universiti Malaysia Terengganu, 21030 Kuala Nerus, Terengganu, Malaysia.; c School of Marine and Environmental Sciences (PPSMS), Universiti Malaysia Terengganu, 21030 Kuala Nerus, Terengganu, Malaysia.; d Institute of Oceanography and Maritime Studies (INOCEM), Kulliyyah of Science, International Islamic University Malaysia Kuantan, Jalan Sultan Ahmad Shah, 25200 Kuantan, Malaysia.; e Department of Marine Science, Kulliyyah of Science, International Islamic University Malaysia Kuantan, Jalan Sultan Ahmad Shah, 25200 Kuantan, Malaysia.

## Abstract

The data available in this repository were gathered from Balok, the only most productive spawning site for horseshoe crabs *Tachypleus gigas* and *Carcinoscorpius rotundicauda* in East Coast of Peninsular Malaysia. The mangrove horseshoe crab, *C. rotundicauda* population and spawning data are available in the first table. The horseshoe crabs were retrieved from Balok River using 11.43 cm mesh size gill nets installed at the river mouth, the confluence and last meander. The arthropods were inspected for damage, abnormality and growth before their release into Balok River, particularly at the site of capture. Sediment samples were retrieved at their spawning grounds to ascertain sediment composition and size classifications which were also processed using Logarithmic Method of Moments. Water parameters like temperature, pH and salinity were also investigated during year 2016. All these information are compiled into the second table and arranged according to the period of data availability. The horseshoe crab catch data of years 2012, 2013, 2014, 2015 and 2016 were made available by artisanal fisher and compiled in the third and fourth table for inter-species comparison.

## Specifications table

**Table:** 

Subject area	*Environmental Science*
More specific subject area	*Ecology*
Type of data	*Table*
How data was acquired	*Multi-parameter probe YSI 556 MPS, mechanical shaker with sieves (4-0.063 mm), Logarithmic Method of Moments for mean, sorting, skewness and kurtosis, 2 mm sieve, measuring tape (sensitive 0.1 cm), analytical balance (sensitive 0.01 kg) and three gill nets (2 m × 200 m × 11.43 cm mesh).*
Data format	*Raw and Analyzed*
Experimental factors	*Water turbidity and current speed during physicochemical parameter measurements, anchoring of gill nets during rising tides, condition of sieves (clean and free from tearing), Carcinoscorpius rotundicauda emergence to spawn at Balok River banks, visual sighting of nests on the shore and sufficient field experience to pint-point horseshoe crab spawning sites.*
Experimental features	*Logarithmic Method of Moments is used to calculate and describe sediment mean, sorting, skewness and kurtosis values.*
Data source location	*Tachypleus gigas spawning area:*
	*Balok Beach (3°56׳15.00"N, 103°22׳35.48"E)*
	*Carcinoscorpius rotundicauda spawning area: Balok River*
	*Site 1: 3°56׳20.30"N, 103°22׳35.40"E (accessible recreation area)*
	*Site 2: 3°56׳26.60"N, 103°22׳27.20"E (inaccessible confluence)*
	*Site 3: 3°56׳39.60"N, 103°22׳20.10"E (upstream meander)*
Data accessibility	*All data are available within this article*
Related research article	*N.A.M. Zauki, B.R. Nelson, B. Satyanarayana, N. Fairuz-Fozi, M.B. Martin, B. Akbar-John, A.J.K. Chowdhury, Citizen science frontiers horseshoe crab population regain at their spawning beach in East Peninsular Malaysia. J. Environ. Manage. 232, 2019, 1012-1020.*

## Value of the data

• The present data informs about gender fractions for juvenile, abnormal and sexually inactive (infested with epibionts) mangrove horseshoe crabs available at Balok River and its estuary.
• By-catch data informs about horseshoe crab (*C. rotundicauda* and *T. gigas*) co-existence, their wild population approximate size and the river carrying capacity which become useful for safeguarding measures.
• Ecological data of the study area, Balok River and its estuary provide insights on sediment nomenclature and water physicochemical properties at the *Carcinoscorpius rotundicauda* spawning site.

## 1 Data

Acquisition of present data is possible because *Carcinoscorpius rotundicauda* nests were containing newly fertilized eggs, adult horseshoe crabs were entangled in the 2 m × 200 m × 11.43 cm (mesh size) gill nets and fishermen logged their horseshoe crab catch from years 2012 to 2016. While *Carcinoscorpius rotundicauda* population structure data are provided (Table 1), sediments from sites containing horseshoe crab nests are also provided to inform about grain sizes, proportions and nomenclatures. These sediment properties were arranged with *Carcinoscorpius rotundicauda* capture and visual inspections as well as Balok River water physicochemical properties like temperature, pH and salinity (Table 2). Importantly, artisanal by-catch data of two horseshoe crab species are made available for comparison and also becomes horseshoe crab wild stock data at Balok River over 5 years (Tables 3 and 4).

**Table 1:** Adult *Carcinoscorpius rotundicauda* conditions and their spawning data at Balok for year 2016.

Horseshoe crab data		Nest (Nos.)	Clutches (Nos.)	Eggs (Nos.)	Male (Nos.)	Female (Nos.)	Amplexus (Nos.)	Juvenile (Nos.)	Infestation (Nos.)	Abnormal (Nos.)
January	S1	0	0	0	3	1	0	0	0	0
February	S2	1	1	78	0	0	0	0	0	0
April	S2	4	4	426	2	0	0	0	0	1
May	S2	1	1	76	0	0	0	0	0	0
June	S1	0	0	0	0	0	0	0	0	0
	S2	1	1	41	23	3	8	2	12 (*F* = 2)	9 (*F* = 3)
	S3	13	13	477	2	0	2	0	3 (*F* = 3)	0
July	S1	0	0	0	0	0	0	0	0	0
	S2	9	9	360	12	0	9	0	7 (*F* = 1)	2 (*F* = 4)
	S3	1	1	86	4	5	3	0	3 (*F* = 3)	3
Aug	S1	14	15	689	23	2	3	1	17	5 (*F* = 5)
	S2	0	0	0	18	3	2	0	11 (*F* = 1)	4 (*F* = 3)
	S3	7	7	255	2	3	12	1	6 (*F* = 5)	7
September	S1	5	7	607	11	3	3	0	15	3 (*F* = 1)
	S2	1	1	78	11	1	5	0	12 (*F* = 2)	3 (*F* = 2)
	S3	4	5	211	3	0	3	0	6 (*F* = 6)	2 (*F* = 2)
October	S1	11	11	539	0	0	1	0	0	1
	S2	0	0	0	1	0	0	0	0	1 (*F* = 1)
	S3	1	1	18	0	0	5	0	4 (*F* = 1)	2 (*F* = 1)
November	S1	11	11	605	3	0	0	0	2	0
	S2	1	1	38	0	0	0	0	0	0
	S3	0	0	0	0	0	1	0	1 (*F* = 1)	0
December	S1	4	4	400	2	2	0	1	2 (*F* = 1)	0
	S2	1	1	53	0	0	0	0	0	0
	S3	1	1	80	0	0	0	0	0	0
	**Total**	**91**	**95**	**5117**	**120**	**23**	**57**	M = 3%	M = 57%, F = 33%	M = 24%, F = 28%

Note: Data for March are not available, the parameters are recorded as numbers (nos.), sampling sites are labelled S1–S3, total yield of juvenile, crabs with infestation and abnormal crabs are indicated by gender (M = male and F = female) and measured as percentage (%) whereas yield of juveniles are only available for male crabs.

**Table 2:** Data consisting of sediment properties and water physicochemical parameters from Balok during year 2016.

**Environmental data**		Sediment												Water		
		Mean (Xϕ)	Sorting (σϕ)	Skewness (SKϕ)	Kurtosis (Kϕ)	Gravel (%)	Sand (%)	Silt & clay (%)	0.063 mm (%)	0.09 mm (%)	0.125 mm (%)	TOC (%)	Nest depth (cm)	Temp. (°C)	pH	Salinity (ppt.)
January	S1	2.3	0.4	−1.2	9.1	0.1	99.9	0.0	0.0	0.3	13.0	0.0	0.0	29.4	6.9	9.8
February	S2	2.4	1.4	−1.1	2.6	1.6	85.6	12.8	20.4	28.8	22.6	0.7	2.0	28.8	7.0	17.4
April	S2	2.6	0.9	−2.1	7.2	0.3	95.3	4.5	3.3	14.2	48.2	1.2	2.8	31.2	7.6	NA*
May	S2	2.3	1.5	−0.9	2.2	0.8	85.0	14.2	17.8	30.9	15.5	1.4	1.0	31.0	6.4	24.2
June	S1	2.4	1.0	−1.4	5.0	0.6	95.7	3.7	6.8	11.8	32.5	0.1	0.0	29.6	6.6	28.2
	S2	2.2	1.5	−0.8	2.2	1.4	87.7	10.9	15.9	29.5	17.3	1.4	2.0	31.4	7.1	27.9
	S3	1.4	1.5	0.2	1.6	2.1	78.7	19.2	12.1	11.8	7.2	2.2	3.0	28.8	6.5	24.7
July	S1	2.5	0.8	−1.9	7.7	0.3	97.1	2.6	5.1	12.6	38.8	0.0	0.0	29.4	6.5	18.0
	S2	2.4	1.4	−1.0	2.4	1.2	86.6	12.3	17.6	28.7	21.8	1.5	2.7	28.7	6.7	16.5
	S3	1.0	1.3	0.5	2.0	2.1	70.6	27.3	2.4	9.3	8.5	2.3	1.5	28.3	6.7	9.5
Aug	S1	2.5	1.1	−1.5	4.5	0.7	93.4	5.9	9.7	19.2	36.2	0.1	2.7	30.7	7.5	28.7
	S2	2.5	1.4	−1.1	2.6	0.3	85.7	13.9	24.9	33.5	11.5	1.4	0.0	30.1	7.4	28.2
	S3	1.8	1.5	−0.2	1.5	0.4	73.2	26.4	14.6	14.8	12.0	2.9	2.9	30.9	6.9	21.5
September	S1	2.6	0.9	−2.1	7.4	0.5	95.1	4.4	7.5	17.6	44.8	0.1	2.6	30.3	6.9	11.6
	S2	2.1	1.5	−0.6	1.6	0.4	72.9	26.8	11.4	34.1	13.7	1.5	2.0	31.0	6.7	7.1
	S3	1.5	1.5	0.1	1.5	2.7	71.1	26.2	12.1	12.7	8.6	2.5	2.2	29.8	6.5	6.7
October	S1	2.5	1.2	−1.3	3.5	0.5	86.3	13.1	15.0	19.3	34.8	0.1	3.5	28.7	6.5	2.0
	S2	2.3	1.4	−1.0	2.4	0.7	83.2	16.2	8.2	32.8	25.8	1.3	0.0	28.9	6.4	6.1
	S3	2.2	1.4	−0.6	1.9	0.4	83.3	16.3	19.3	19.4	13.0	2.3	3.0	28.7	6.3	2.4
November	S1	2.5	1.0	−1.8	6.6	0.8	95.7	3.5	8.1	21.4	40.3	0.1	3.0	28.7	6.6	4.7
	S2	2.6	1.2	−1.4	3.8	0.3	90.0	9.7	16.4	37.4	21.5	1.5	3.0	28.5	6.4	6.4
	S3	1.8	1.2	−0.4	2.3	0.6	86.5	12.9	7.2	11.6	21.4	2.5	0.0	28.3	6.5	2.8
December	S1	2.6	0.9	−1.9	6.6	0.4	95.3	4.3	6.4	21.4	43.4	0.1	4.3	27.0	6.7	1.8
	S2	2.7	1.1	−1.8	5.3	0.3	94.6	4.6	11.7	40.3	28.5	1.3	3.0	27.7	6.6	1.3
	S3	2.4	1.1	−1.0	3.2	0.3	96.0	3.7	10.2	20.2	32.2	2.9	4.0	27.5	7.3	1.1

Note: Data for March are not available, sampling sites are labelled S1–S3, unavailable data are indicated with NA*, Logarithmic Method of Moments denote mean, sorting, skewness and kurtosis as phi (*ϕ*), sediment fractions (gravel, sand, silt and clay) are measured as percentage (%), grain sizes (0.063–0.125 mm) are measured as percentage (%), nest depth measured as centimetre (cm), water temperature measured as Celsius (°C) and salinity measured as parts-per-thousand (ppt).

**Table 3:** Adult *Tachypleus gigas* catch data by artisanal fisher of Balok between years 2012 and 2016.

*Tachypleus gigas* catch data	2012		2013		2014		2015		2016	
	Hand (Nos.)	Net (Nos.)	Hand (Nos.)	Net (Nos.)	Hand (Nos.)	Net (Nos.)	Hand (Nos.)	Net (Nos.)	Hand (Nos.)	Net (Nos.)
January	6	16	6	18	5	21	4	25	9	23
February	6	15	6	17	6	18	5	22	7	22
March	7	16	8	18	5	16	4	19	10	20
April	4	10	5	12	4	12	4	13	7	16
May	9	20	10	23	9	23	11	22	13	24
June	10	22	11	25	7	25	8	24	10	28
July	6	21	7	24	6	21	7	20	9	21
August	6	11	7	13	3	18	4	17	5	19
September	5	7	6	8	0	15	0	14	4	15
October	2	5	2	6	2	6	3	7	0	6
November	4	12	4	14	5	12	6	16	2	18
December	6	13	6	15	6	16	7	21	5	19
**Total**	**71**	**168**	**77**	**194**	**58**	**203**	**62**	**219**	**81**	**231**

Note: Yield of horseshoe crabs either by hand-catching or net-catching are counted as numbers (Nos).

**Table 4:** Adult *Carcinoscorpius rotundicauda* catch data by artisanal fisher of Balok between years 2012 and 2016.

*Carcinoscorpius rotundicauda* catch data	2012		2013		2014		2015		2016	
	Hand	Net	Hand	Net	Hand	Net	Hand	Net	Hand	Net
January	4	4	4	5	2	7	2	8	3	11
February	4	3	4	3	1	4	1	5	0	3
March	2	3	2	3	2	2	2	2	3	2
April	4	2	5	2	3	2	3	2	1	5
May	6	4	7	5	4	3	5	3	3	6
June	5	6	6	7	6	4	7	4	5	5
July	3	8	3	9	4	2	5	2	2	6
August	3	5	3	6	1	5	1	5	3	9
September	0	4	0	5	1	3	1	3	2	10
October	1	2	1	2	2	8	3	9	4	7
November	2	2	2	2	3	8	3	10	5	8
December	3	0	3	0	5	7	6	9	4	12
**Total**	**37**	**43**	**40**	**50**	**34**	**55**	**38**	**62**	**35**	**84**

Note: Yield of horseshoe crabs either by hand-catching or net-catching are counted as numbers (Nos).

## 2 Experimental design, materials, and methods

Field visits to Balok River and its estuary, Balok Beach were carried out from January to December 2016 [1]. Gill nets with 2 m × 200 m (size) × 11.43 cm (mesh size) measurements were installed an hour before rising tides at the river mouth, Balok River confluence and upstream meander. Horseshoe crab nests were searched along Balok River banks during low tide periods [2]. It was this time that *Carcinoscorpius rotundicauda* nest and eggs were counted, sediment samples collected and water physicochemical measurements like temperature, pH and salinity recorded. Simultaneously, meet with fisher community provided us with two horseshoe crab species by-catch data between years 2012 and 2016. At the laboratory, 100 g sediments were oven dried for three days, placed onto series of sieves (4–0.063 mm) and shaken on a mechanical shaker. Then, the sediment fractions in each sieve were measured to elucidate sand, gravel and silty and clay fractions on an analytical balance. Yield of sediment fractions were computed into mean, sorting, skewness and kurtosis annotations using Logarithmic Method of Moments calculations in Microsoft Excel 2013 [3].
