# Supplementary material for: Horseshoe crab bio-ecological data from Balok, East Coast Peninsular Malaysia
Source: Data Brief. 2018 Dec 13;22:458–63. doi: 10.1016/j.dib.2018.12.027 (PMC6312785; doi:10.1016/j.dib.2018.12.027)
Supplement: Supplementary file 1 — Supplementary material [file mmc1.doc]

Conflict of Interest and Authorship Conformation Form

Please check the following as appropriate:

( / ) All authors have participated in (a) conception and design, or analysis and interpretation of the data; (b) drafting the article or revising it critically for important intellectual content; and (c) approval of the final version.

( / ) This manuscript has not been submitted to, nor is under review at, another journal or other publishing venue.

( / ) The authors have no affiliation with any organization with a direct or indirect financial interest in the subject matter discussed in the manuscript

Author’s name Affiliation

1) Nurul Ashikin Mat Zauki Mangrove Research Unit, Institute of Oceanography and Environment, Universiti Malaysia Terengganu

2) Behara Satyanarayana Mangrove Research Unit, Institute of Oceanography and Environment, Universiti Malaysia Terengganu

3) Nur Fairuz Fozi Mangrove Research Unit, Institute of Oceanography and Environment, Universiti Malaysia Terengganu

4) Bryan Raveen Nelson Institute of Tropical Biodiversity and Sustainable Development, Universiti Malaysia Terengganu

5) Melissa Beata Martin School of Marine and Environmental Sciences, Universiti Malaysia Terengganu

6) Akbar John Bavajohn Institute of Oceanography and Maritime studies, Kulliyyah of Science, International Islamic University Malaysia

________________________________________________________________________

7) Ahmed Jalal Khan Chowdhury Department of Marine Science, Kulliyyah of Science, International Islamic University Malaysia

________________________________________________________________________
